# Demonstration of single crystal growth via solid-solid transformation of a glass

**DOI:** 10.1038/srep23324

**Published:** 2016-03-18

**Authors:** Dmytro Savytskii, Brian Knorr, Volkmar Dierolf, Himanshu Jain

**Affiliations:** 1 Materials Science and Engineering Department, Lehigh University, Bethlehem, PA 18015, USA; 2 Physics Department, Lehigh University, Bethlehem, PA 18015, USA

## Abstract

Many advanced technologies have relied on the availability of single crystals of appropriate material such as silicon for microelectronics or superalloys for turbine blades. Similarly, many promising materials could unleash their full potential if they were available in a single crystal form. However, the current methods are unsuitable for growing single crystals of these oftentimes incongruently melting, unstable or metastable materials. Here we demonstrate a strategy to overcome this hurdle by avoiding the gaseous or liquid phase, and directly converting glass into a single crystal. Specifically, Sb_2_S_3_ single crystals are grown in Sb-S-I glasses as an example of this approach. In this first unambiguous demonstration of an all-solid-state glass → crystal transformation, extraneous nucleation is avoided relative to crystal growth via spatially localized laser heating and inclusion of a suitable glass former in the composition. The ability to fabricate patterned single-crystal architecture on a glass surface is demonstrated, providing a new class of micro-structured substrate for low cost epitaxial growth, active planar devices, etc.

Inorganic solids generally exist in either a disordered glassy, a polycrystalline ceramic, or a fully ordered single crystal state. A transformation from glass to ceramic is achieved readily by heating the former to a particular temperature that inevitably leads to nucleation of many crystals^1^,^2^. In producing a single crystal, the creation of multiple nuclei must be avoided. For this reason, most single crystals are produced today not by solid-solid, but by liquid-solid transformation^3^ in which the formation of extraneous nuclei during the growth of the initially formed nucleus is unstable in the surrounding liquid phase. However, there is a serious drawback of single crystal growth from melts: such methods are not useful for fabricating crystals of compositions that decompose, transform to some undesirable phase, or melt incongruently on heating^4^. Consequently, it has been extremely difficult or impossible to grow single crystals of many complex oxides such as high Tc superconductors^5^, organometallic halide perovskites for solar cells of exceptional efficiency^6^,^7^, etc. This lack of high quality crystals is identified as a critical limitation to the progress of materials by design paradigm^8^. For these materials, elevated temperatures and melting need to be avoided. In principle, this can be achieved by a strategy, in which the glassy material is heated locally by a laser to just its crystallization temperature (T_x_), which is well below the melting temperature. Using glass as a precursor and a focused laser as a localized heating source, offers the combined advantages of low cost, of allowing broad composition ranges, and of easy formability of single crystals in complex shapes including wires or films. Furthermore, they enable a new materials platform comprising of a ‘single crystal architecture in glass (SCAG)’, in which the single crystal of arbitrary shape can be an active phase with properties such as second order optical nonlinearity, ferroelectricity, pyroelectricity, piezoelectricity, etc., that are not possible in the isotropic structure of glass. Consequently, this method for converting glass to single crystal can have a transformational impact on multiple technologies.

The concept of glass → single crystal transformation could not be realized in the past due to concurrent nucleation at multiple sites, which ultimately produced polycrystalline glass-ceramic instead of single crystal^1^,^2^. On the other hand, basic feasibility of SCAG fabrication has been demonstrated recently at or near the surface of glass by Komatsu *et al*.^9^, as well as deep inside the glass by Stone *et al*.^10^ using continuous wave (CW) and femtosecond (fs) lasers, respectively. However, in all these reports, the single crystal is formed during cooling of molten material that is produced in the vicinity of the laser focus^9^,^10^. This process does not have the desired advantages of a solid-state transformation of glass → single crystal, and very much resembles the traditional floating zone crystal growth from the melt^11^.

Although epitaxial conversion of solid amorphized surface film of semiconductors (such as from ion implantation) back to single crystal^12^, and growth within a polycrystalline solid through seeded conversion have been reported^13^, there is no evidence in the literature showing all solid- state conversion of bulk solid glass to a single crystal. In contrast to these works, only the single crystal growth that occurs directly from solid glass offers the possibility of SCAG for crystal compositions that melt incongruently, decompose on heating to the melting temperature (T_m_), or for which the desired crystalline phase is unstable at temperatures between T_x_ and T_m_. Moreover, the underlying science and mechanism of the phase transformation from melt-to-crystal vis-à-vis directly from glass → crystal are fundamentally different.

So, how can a glass → single crystal transformation be achieved? The basic premise of fabricating a single crystal is simple: establish only one nucleus and then help it grow to the desired dimensions. It is implicit that for this to happen the experimental conditions should inhibit the formation of any other competing nuclei while the initially nucleated crystal grows. This is most readily accomplished by destabilizing nucleation in the vicinity of the growing crystal by maintaining the temperature slightly <T_m_, in the metastable Ostwald-Miers supercooled zone^1^. In this temperature range no nucleation occurs (see Fig. 1a), and an external seed crystal is utilized such as in Czochralski method^3^.

On the other hand, when one relies on spontaneous nucleation such as in the case of glass devitrification, multiple crystals grow simultaneously resulting in a polycrystalline ceramic. Since the temperature of nucleation onset (T_n_) is always lower than T_x_^1^,^2^, unwanted nuclei are always likely to form and remain stable around the heated region^14^ as shown schematically in Fig. 1. Notwithstanding, we have devised a strategy that allows us to grow a single crystal using direct crystallization of glass, which involves heating it from ambient to T_x_ (see Fig. 1). Nucleation is a stochastic process so that its overall probability depends on the volume of the heated region and the time. Heating with a focused laser can limit the volume of glass so that only one nucleus is allowed to form, which is then grown quickly into a single crystal. We show that unwanted additional nucleation can be avoided by decreasing the volume of the heated region and growing the crystal by moving the laser beam at a sufficiently fast rate such that there is no time for forming extraneous crystals.

To validate our strategy and demonstrate proof-of-concept, it is most appropriate to begin with a composition that is within the glass-forming region but not too far from the boundary where crystallization is unavoidable. If the glass is highly stable, the probability of nucleation, especially homogeneous nucleation, and hence controlled laser crystallization is too low to test the hypothesis in a reasonable time. On the other hand, if the composition crystallizes too easily, precise observation of the crystallization process, especially single-crystal formation would become difficult. Further, for experimental convenience the glass should be able to absorb readily available laser light in a sufficiently deep region of the sample. A laser that is strongly absorbed just in the very top surface layer (<1 μm) is not desirable, as the nucleation becomes relatively improbable and the crystal growth is not as well controlled. The bandgap of most chalcogenide glasses falls into the visible to near-infrared spectral region, so that light from red lasers is absorbed efficiently and no additional dopants are required in contrast to oxide glasses^9^,^15^. Changing the wavelength of the laser diode allows altering the corresponding absorption cross-section conveniently, which would facilitate modification of the temperature profile within the sample, providing a useful tool for optimizing crystal nucleation/growth dynamics.

For all the above-mentioned reasons, we selected Sb_2_S_3_ composition as a test example. This simple binary composition belongs to technologically important A_2_B_3_ type chalcogenides (A = As, Sb, Bi; B = S, Se, Te), which have been investigated due to their attractive physical and chemical properties^16^,^17^,^18^,^19^. Consequently, their basic physical, thermodynamic and chemical properties have been determined and are readily available in the literature. Among possible choices, antimony trisulfide (Sb_2_S_3_) is particularly attractive because of its interesting ferroelectric properties^20^ and potential practical applications in solar cells, microwave devices, switching sensors, thermoelectric and optoelectronic devices^21^,^22^,^23^,^24^,^25^. To exemplify the impact of the proposed new strategy of single crystal fabrication, we note that this material burns in air at ~300 °C, and loses sulfur preferentially upon heating to high temperature in an inert atmosphere^26^,^27^,^28^. Therefore, it is practically impossible to obtain its stoichiometric single crystal by starting from melt using conventional methods.

## Results

### 1-D single crystal fabrication

The success of the space selective laser-induced heating for transforming glass into single crystal is evident from the results shown in Fig. 2, using the example of Sb_2_S_3_ glass. We employed a diode laser with wavelength (λ) of 639 nm, which is focused to a few μm on the surface, and its intensity is gradually increased from 0 to 50 μW/μm^2^ in 5 s and then maintained at this value. The first sign of a crystal is observed 2 s thereafter. Within 20 s it reaches the equilibrium dimensions as seen in Fig. 2. The uniform color of inverse pole figure (IPF) maps obtained from electron backscatter diffraction (EBSD) analysis confirms that the Sb_2_S_3_ glass transformed into a single crystal dot by laser heating (see Supplementary video SV1 for the details).

As the laser beam is subsequently moved laterally across the surface at a rate of 1 μm/s, the growth of the initial dot follows the laser, forming a single crystal line of Sb_2_S_3_ as seen in Fig. 2. The orientation IPF maps for both the dot and line exhibit the same color, which confirms that the whole structure is a single crystal of Sb_2_S_3_ (Fig. 2c,d).

In order to extend our approach to other materials systems, we further suppressed unwanted nucleation by adding a glass-forming component. This additional strategy can have broad applicability through appropriate choice of glass composition. For its validation, we repeated the above experiments on homogeneous 16SbI_3_–84Sb_2_S_3_ glass wherein the addition of 16% SbI_3_ makes glass formation easier and nucleation more difficult relative to Sb_2_S_3_^29^. Nevertheless,when heated with a laser beam only Sb_2_S_3_ crystalline phase precipitates out either through the evaporation of SbI_3_ in the heated zone or enrichment of the region around the growing crystal with iodine and antimony. In either case, nucleation in front of the growing crystal is suppressed relative to crystal growth. Figure 3a shows typical morphology of the initial dot (D1), which was induced by laser beam above a minimum threshold intensity of 65 μW/μm^2^. The energy dispersive spectroscopy (EDS) analysis maps (Fig. 3e) for this region show deficiency of iodine, which indicates SbI_3_ evaporation under sustained heating by the laser beam. This change in composition increases the concentration of Sb_2_S_3_ and stimulates the formation of its crystal in shallow crater.

To assess the tendency of nucleation relative to the growth of Sb_2_S_3_ crystal in 16SbI_3_–84Sb_2_S_3_ glass, the laser was turned off after forming the single crystal line L1. It took less than 1 s to form a new dot next to the previously formed line L1, compared to ~43 s needed to form the initial dot D1. Therefore, the crystal line can be grown indefinitely using the previously created crystal as a seed.

### 2-D single crystal fabrication

Having demonstrated the feasibility of solid glass → single crystal transformation and the ability to fabricate single crystal lines by eliminating extraneous nucleation, we turned our attention to the realization of 2D crystals, further enhancing the usefulness of solid state crystal growth as a SCAG. Based on Fig. 4, which shows a 2D crystal of Sb_2_S_3_ grown on the surface of 16SbI_3_–84Sb_2_S_3_ glass, it is indeed possible to ‘stitch’ successive lines together to form a 2D crystal. In this approach^30^, the laser is moved from the initial dot D1 in *X*- direction at 20 μm/s, and the first Sb_2_S_3_ single crystal, L1, grows without introducing additional nuclei. To obtain the second line, the end of first line is used as the seed. Laser exposure for the second and subsequent dots (D2–D7) was reduced to 15 s compared to 60 s for D1. Then the second line was written anti-parallel to and overlapping the first line. The subsequent laser- written crystal lines were written similarly, overlapping with the previous line by slightly more than half the width of the previous line. The result is a 2D planar single-crystal structure made via solid-solid transformation, with *c-*axis orientation normal to the laser scanning direction for the whole area as shown by the EBSD maps in Fig. 4. Each crystal in these dots (D2–D7) and subsequent lines maintains the same orientation. The neighboring lines merge and form the 2D single crystal structure.

The reproducibility of the observations reported here is excellent as established from experiments on a few tens of dots and lines, and several 2D structures using optimal laser irradiation parameters (power, focus position relative to surface, scanning rate, etc.).

## Discussion

Previous attempts of crystallization of amorphous Sb_2_S_3_ films, which did not follow the strategy developed in the present work, produced only polycrystalline structures^31^,^32^. The authors of these investigations used argon laser with a spot size of 400 μm diameter, and no attempt was made to maintain the temperature below the melting temperature. Sb_2_S_3_ does not form glass easily; it requires very rapid cooling of the melt to form bulk glass, as in this study, or vacuum deposition of its vapor phase to form thin amorphous films prepared by Arun *et al*.^31^,^32^. Therefore, in their work the probability of extraneous nucleation was too high to yield a single-crystal upon heating. This challenge of unwanted nucleation could be successfully overcome by decreasing significantly the heated volume with a finely focused laser beam, and addition of SbI_3_ as a glass stabilizer.

**Figure 1:**
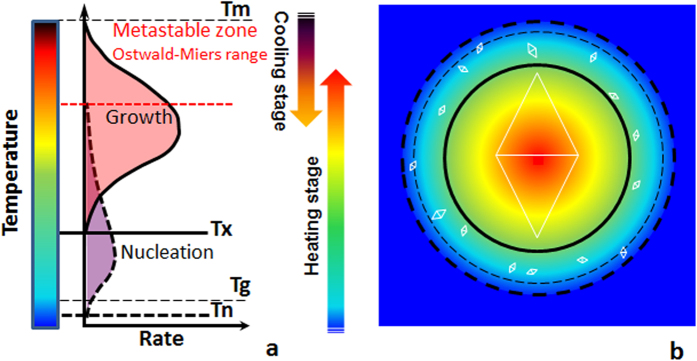
Temperature dependence of nucleation and crystal growth rates in glass-forming systems and its relationship to laser crystallization. (**a**) Prototypical temperature dependence vs. nucleation and growth rate curves. (**b**) Schematic of the CW laser induced temperature fields in the focal spot at the glass surface, with the temperature lines from (**a**) representing the lower limits of nucleation and crystal growth temperature range. Small rhombuses indicate the region of unwanted nuclei, while larger rhombus indicates the region of single crystal formation. T_m_, T_x_, T_n_ and T_g_ indicate the temperatures of melting, crystal growth onset, nucleation onset and glass transition.

**Figure 2:**
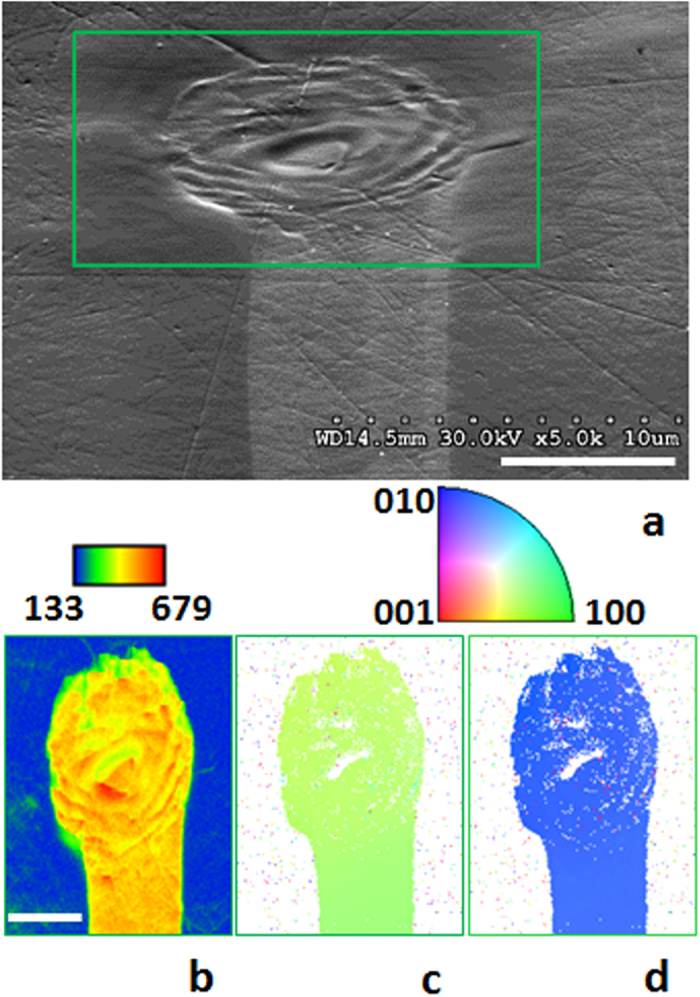
Laser-induced formation of Sb_2_S_3_ single crystal line on the surface of Sb_2_S_3_ glass. A laser-induced dot created on the surface of Sb_2_S_3_ glass by slowly ramping the laser power density from 0 to 50 μW/μm^2^ in 5 s, followed by steady exposure for 60 s, and its extension into a straight line by moving the laser spot at the speed of 1 μm/s. Scale bar corresponds to 5 μm. (**a**) SEM image, (**b**) colored IQ map, and orientation IPF maps with reference vector along surface normal (**c**) and along in-plane direction (**d**), respectively. EBSD mapping is collected using 70^o^- inclined geometry, which in the case of rough sample surface results in a “shadow” region inaccessible to the probe electron beam. The white-colored regions on IPF maps inside of the dot correspond to such “shadow” regions.

There are two independent key observations pertaining to the premise of this Report, which prove that the glass → single crystal transformation occurs here entirely in the solid state. First, scratches that were present on the glass surface before laser irradiation (seen in Fig. 2c,d, most clearly in the region of the line) persist through the crystallization process, indicating that the nucleation and growth processes occur without forming a melt that would have altered the surface morphology. Second, the *in situ* observation of the crystal growth process (see Supplementary video SV1) demonstrates that the crystallization occurs at the leading, not the trailing edge of the laser beam. The former region represents the region being heated from ambient to crystallization temperature, while the latter represents the region cooled to ambient from the crystallization temperature. This is a direct indication that the glass transforms into single crystal upon its heating, and not during the cooling of the melt that would have happened at the trailing edge of the laser spot^14^. Thus, these results provide the first unequivocal proof-of-principle that it is possible to transform a glass into single-crystal by heating to crystallization onset temperature (T_X_), rather than by the usual crystal growth processes via cooling to the crystallization temperature from above the melting point (Fig. 1).

**Figure 3:**
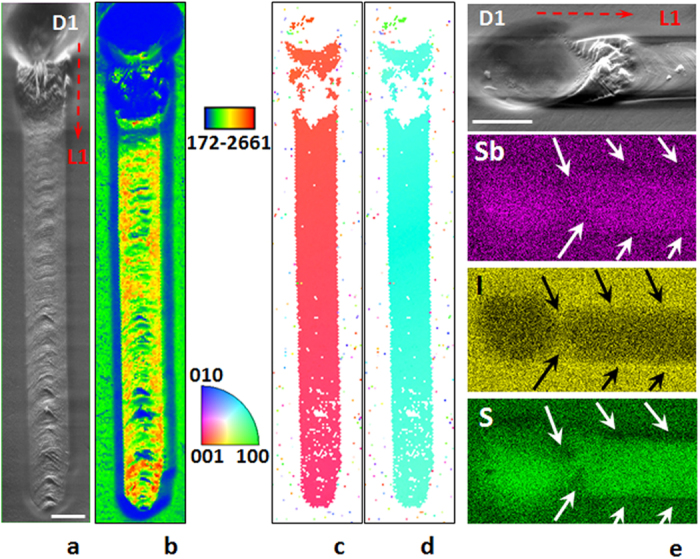
Laser-induced formation of Sb_2_S_3_ single crystal line on the surface of 16SbI_3_–84Sb_2_S_3_ glass. A single-crystal dot (D1) was created by slowly ramping the power density from 0 to 90 μW/μm^2^ in 5 s, followed by steady exposure for 60 s. Scale bar corresponds to 5 μm. (**a**) SEM image, (**b**) IQ and colored orientation IPF maps with reference vectors (**c**) along surface normal and (**d**) an in-plane direction for the single-crystal line; (**e**) SEM image and EDS color maps for sulfur, iodine and antimony in the dot D1 → line L1 transition region. The rough sample surface in part of the dot and beginning of the line do not show an EBSD signal due to the “shadow” effect for the probe electron beam.

**Figure 4:**
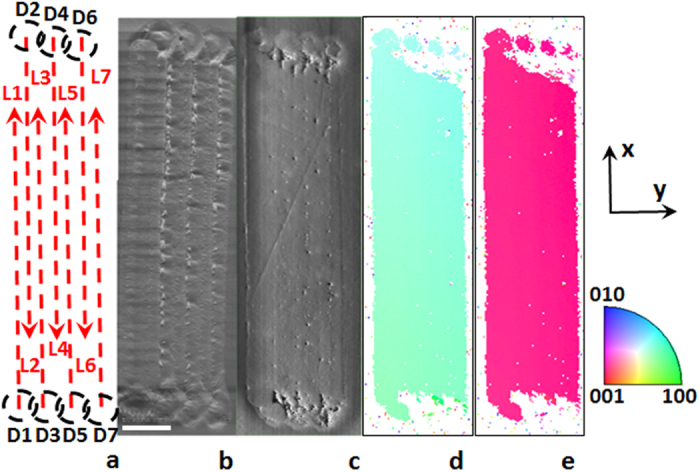
2D laser-induced single-crystal architecture fabricated by ‘stitching’ seven lines, L1-L7. First, a seed dot D1 and line L1 were created on the surface of 16SbI_3_–84Sb_2_S_3_ glass, as in Fig. 3. New seed dots (D2–D7) were formed from previous lines, and then used to grow new lines (L2–L7), correspondingly. To form dots D2–D7 the beam was shifted laterally in the *y*- direction by 3 μm and the exposure was reduced to 15 s. (**a**) Plan-view; SEM images (**b**) before and (**c**) after repolishing; and orientation IPF maps with reference vectors (**d**) along surface normal and (**e**) the in-plane direction, respectively. Scale bar corresponds to 10 μm.

As for the lines fabricated in Sb_2_S_3_ glass, the crystallization also occurs at the leading edge of the laser-heated region (see Supplementary video SV2), which confirms the growth of single- crystal Sb_2_S_3_ line by the solid state transformation of 16SbI_3_–84Sb_2_S_3_ glass during heating. The relatively small volume contraction of the line compared to the initial dot in Fig. 3 suggests that the crystal growth occurs by the redistribution of Sb, S and I atoms during growth rather than evaporation of SbI_3_. The same is also indicated by the depletion of S and enrichment of I just outside the line (arrows in Fig. 3e).

In conclusion, we have demonstrated that indeed it is feasible to fabricate SCAG via a completely solid-state transformation. As proof-of-principle, successful examples of 1D and 2D Sb_2_S_3_ single-crystal structures are produced on the surface of xSbI_3_–(1 − x)Sb_2_S_3_ glass by employing a strategy, which relies on eliminating extraneous nucleation relative to crystal growth via space localized laser heating below T_m_, and adding suitable glass former. The new method offers the opportunity to obtain single crystals that may decompose, melt incongruently or undergo phase transformation between the crystallization and melting temperature of the glass.

## Methods

### Glass preparation

The glasses were made following the ampule quenching method previously developed for the Sb-S-I system^33^. To make Sb_2_S_3_ samples, which does not form glass easily, the melt cooling rate was increased by limiting quartz ampules to 1 mm inside diameter (ID) and 10 μm wall thickness. 16SbI_3_–84Sb_2_S_3_ glass was prepared using ampules with 11 mm ID and wall thickness 1 mm. X-ray diffraction analysis of the as-quenched samples confirmed their amorphous state. For details of glass fabrication and its characterization, please see Supplementary Information, Figs S1–S3. The samples for laser-induced treatments were polished using metallographic techniques.

**Laser-induced crystallization.** The intensity of the fiber-coupled 639 nm diode laser (LP639- SF70, ThorLabs) used for crystallization, was modulated by an analog voltage (ILX Lightwave LDX-3545 Precision Current Source). The beam was focused onto the sample by a 50x, 0.75NA microscope objective. The sample was placed in a flowing nitrogen environment on a custom- built stage, which could be translated independently in the x-, y-, and z-directions. Flow of nitrogen eliminated oxidation of Sb_2_S_3_ crystals, which was observed in air environment. A CCD camera monitored the sample *in-situ*, while LabView software controlled the laser intensity, and the movement of the stage. A detailed description of laser crystallization system is presented in Supplementary Information, Fig. S4.

### Materials characterization

The laser-irradiated regions were analyzed by a scanning electron microscope (SEM, Hitachi 4300 SE) in water vapor environment to eliminate charging effects. The chemical compositions were determined at multiple locations on each sample by EDS detector attached to SEM, using the EDAX-Genesis software. Local crystallinity and orientation were determined by EBSD with Kikuchi patterns collected by a Hikari detector within the SEM column. EBSD pattern scans were collected and indexed using TSL OIM Data Collection software, whereas Orientation Imaging Microscopy Analysis software yielded image quality, pole figure and inverse pole figure maps^34^.

## Additional Information

**How to cite this article**: Savytskii, D. *et al*. Demonstration of single crystal growth via solid-solid transformation of a glass. *Sci. Rep.* **6**, 23324; doi: 10.1038/srep23324 (2016).

## Supplementary Material

Supplementary Information

Supplementary Video SV1

Supplementary Video SV2
