# Supplementary material for: Demonstration of single crystal growth via solid-solid transformation of a glass
Source: Sci Rep. 2016 Mar 18;6:23324. doi: 10.1038/srep23324 (PMC4796903; doi:10.1038/srep23324)
Supplement: Supplementary Information [file srep23324-s1.pdf]

Supplementary information

"Demonstration of single crystal growth via solid-solid transformation of a glass"

Dmytro Savytskii[1], Brian Knorr[2], Volkmar Dierolf[2] and Himanshu Jain[1]*

[1]Materials Science and Engineering Department, Lehigh University, Bethlehem, PA 18015, USA

[2]Physics Department, Lehigh University, Bethlehem, PA 18015, USA

*E-mail: h.jain@Lehigh.EDU

Supplementary information contents:

Supplementary Video SV1

Supplementary Video SV2

Supplementary Methods and Figures S1-S4

Preparation of Sb_2S_3 glass sample

To obtain fast cooling rates, which are necessary for stoichiometric Sb_2S_3 glasses located outside the normal glass-forming region of the Sb-S-I system, the inner diameter of the ampoules was reduced to 1 mm. The batch of elemental powders was heated to 730 °C and held for 12 h. Then the ampoules containing reacted melt were slowly cooled to 650 °C and quenched in cold water to form glass (Fig. S1, a). The sample together with ampoule was glued to SEM holder and then grinded and polished with grit sizes down to 0.1 μm directly on this holder (Fig. S1, b). X-ray powder diffraction (XRD) analysis of the as-quenched glasses confirmed their amorphous state (see below).

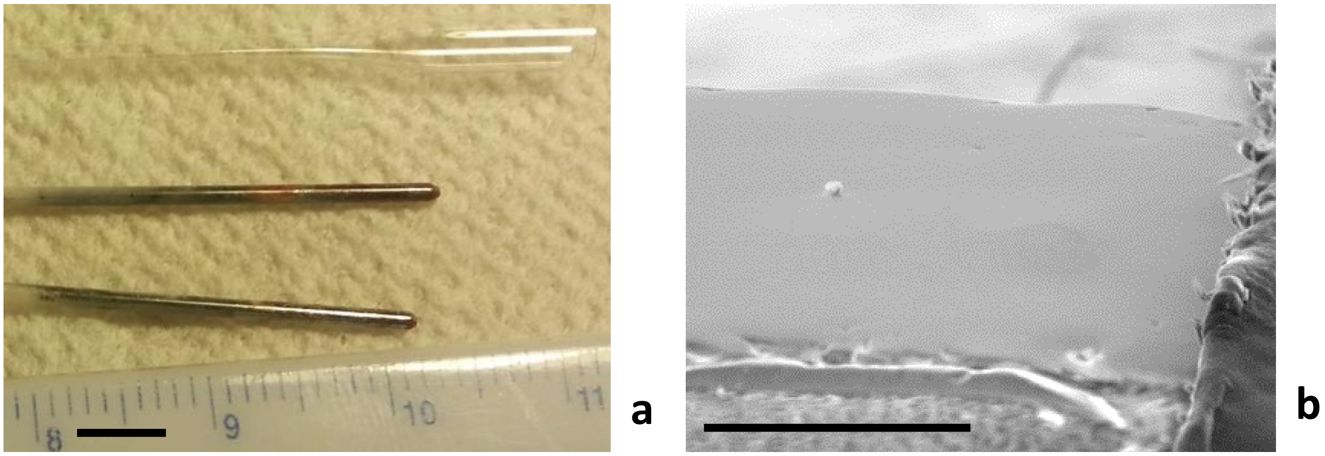

Figure S1. Sb_2S_3 glass: (a) as synthesis in ampule and (b) after polishing Scale bars correspond to 5 and 0.5 mm, correspondingly.

Crystallization propensity

The exothermal peak in differential scanning calorimetry (DSC) measurements on the Sb_2S_3 glass powder with average size 0.06–0.18 mm established crystallization rate maximum at ~217°C. For convenient reference, the DSC plot for the powder heated at 10K/min is shown in Fig. S2. The trace for particles shows only one strong exothermal peak at a temperature close to

T_g. Apart from Sb_2S_3, no other crystalline phase was detected in XRD patterns for the sample after DSC measurements (Fig. S3).

Two partially overlapping DSC peaks (approximately at ~215°C and ~230°C) were observed for the $16SbI_3$–$84Sb_2S_3$ glass powder with 0.06-0.18 mm size particles at low heating rate 3K/min. For glass particles with 0.5-0.7 mm size, the low temperature peak at ~215°C disappeared, but another peak appeared in the high temperature range at ~255°C (Fig. S2). Only one, but wide, DSC peak is observed for powders with different particle sizes when using high heating rate - 10K/min. Similar to the stoichiometric SbSI glass, the SbSI phase in the present composition forms by two parallel (1D and 3D) crystallization mechanisms[1,2]. In contrast to SbSI phase, the temperature of crystallization of Sb_2S_3 phase does not depend on the size of the glass particles[2]. Then for the $16SbI_3$–$84Sb_2S_3$ glass, the peak at ~230°C can be assigned to crystallization of Sb_2S_3 phase.

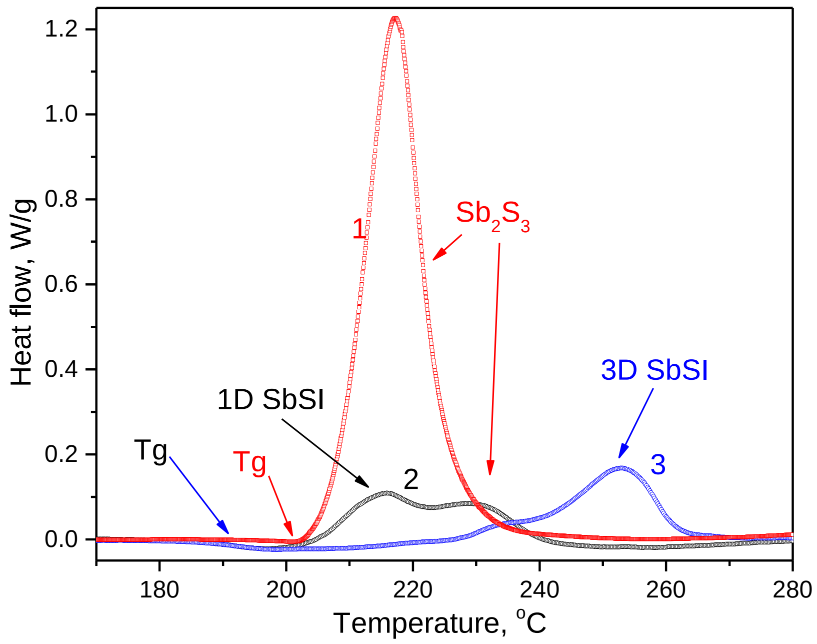

Figure S2. DSC data collected at 10 K/min heating rate on (1) Sb_2S_3 and (2) 16SbI_3–84Sb_2S_3 glass powders of particle size 0.06-0.18 mm . The data for 16SbI_3–84Sb_2S_3 glass powder of particle size 0.5-0.7 mm and collected at 3 K/min heating rate is plot 3.

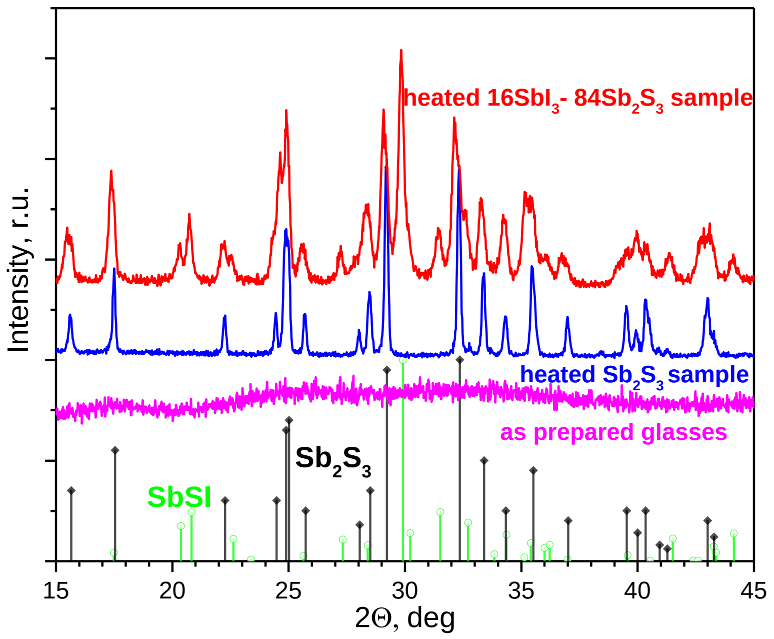

Figure S3. X-ray powder diffraction patterns for as-prepared glass and after heating to 300°C. Vertical lines correspond to reflection positions for crystalline Sb_2S_3. According to ICCD database, card #00-006-0474, space group is Pbnm (62) with lattice parameters a=11.229; b=11.310; c=0.3839 nm, and for crystalline SbSI, the space group is Pnma (62) with lattice parameters: a=0.8533; b=1.0147; c=0.4107 nm.

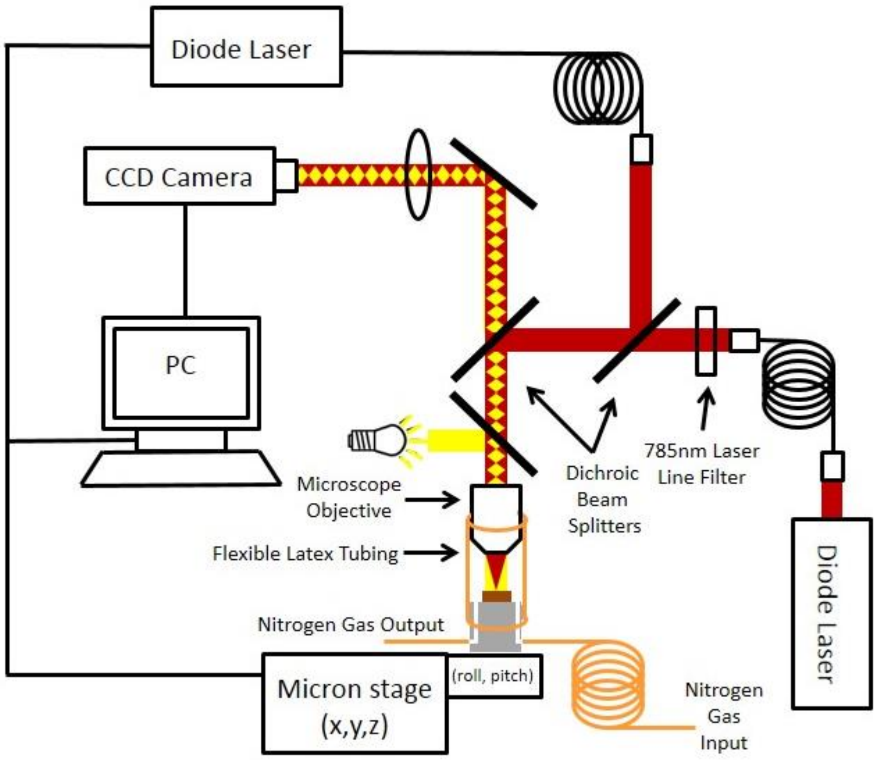

Figure S4. Laser writing setup.

The schematic arrangement of laser writing system is shown in Fig. S4. As the 639 nm light is absorbed by the surface layer of the glass, the spot temperature strongly depends on the power density, which can be manipulated by adjusting the laser intensity, as well as the focus position of the laser beam with respect to the sample surface. The laser beam was defocused relative to the polished surface of the glass sample such that the irradiated area was 7 μm in diameter. Under this condition, the crystalline dots were formed by slowly ramping the power density from zero to 20-105 $\mu W/\mu m^2$ over a period of 5 seconds. Upon reaching the desired power density, the laser was held at the same power for an additional time.

Supplementary Video

Video VS1. Laser-induced formation of Sb_2S_3 single crystal dot and line on the surface of Sb_2S_3 glass. A laser-induced dot created on the surface of Sb_2S_3 glass by slowly ramping the laser

power density from 0 to 50 μW/μm^2 in 5s, followed by steady exposure for 60s, and its extension into a straight line by moving the laser spot at the speed of 1 μm/s.

Video VS2. Laser-induced formation of Sb_2S_3 single crystal dot and line on the surface of $16SbI_3$–$84Sb_2S_3$ glass. A single-crystal dot was created by slowly ramping the power density from 0 to 90 μW/μm^2 in 5s, followed by steady exposure for 60s. Dot was extend into a straight line by moving the laser spot at the speed of 20 μm/s with the power density 90 μW/μm^2.

Supplementary References

[1] Savytskii, D., et. al. Crystallization of stoichiometric SbSI glass. *J. Am. Ceram. Soc*., **97,** 198-205 (2014).

[2] Savytskii, D., Atwater, K., Dierolf, V., Jain, H. Formation of ferroelectric phases in Sb-S-I glasses. *J. Am. Ceram. Soc.*, **97,** 3458–3462 (2014).
